# Urban Tree Species Show the Same Hydraulic Response to Vapor Pressure Deficit across Varying Tree Size and Environmental Conditions

**DOI:** 10.1371/journal.pone.0047882

**Published:** 2012-10-31

**Authors:** Lixin Chen, Zhiqiang Zhang, Brent E. Ewers

**Affiliations:** 1 Key Laboratory Soil and Water Conservation and Desertification Combating, Ministry of Education, College of Soil and Water Conservation, Beijing Forestry University, Beijing, People's Republic of China; 2 Program in Ecology, Department of Botany, University of Wyoming, Laramie, Wyoming, United States of America; Brigham Young University, United States of America

## Abstract

**Background:**

The functional convergence of tree transpiration has rarely been tested for tree species growing under urban conditions even though it is of significance to elucidate the relationship between functional convergence and species differences of urban trees for establishing sustainable urban forests in the context of forest water relations.

**Methodology/Principal Findings:**

We measured sap flux of four urban tree species including *Cedrus deodara*, *Zelkova schneideriana*, *Euonymus bungeanus* and *Metasequoia glyptostroboides* in an urban park by using thermal dissipation probes (TDP). The concurrent microclimate conditions and soil moisture content were also measured. Our objectives were to examine 1) the influence of tree species and size on transpiration, and 2) the hydraulic control of urban trees under different environmental conditions over the transpiration in response to VPD as represented by canopy conductance. The results showed that the functional convergence between tree diameter at breast height (DBH) and tree canopy transpiration amount (*E*_c_) was not reliable to predict stand transpiration and there were species differences within same DBH class. Species differed in transpiration patterns to seasonal weather progression and soil water stress as a result of varied sensitivity to water availability. Species differences were also found in their potential maximum transpiration rate and reaction to light. However, a same theoretical hydraulic relationship between *G*_c_ at VPD = 1 kPa (*G*_cref_) and the *G*_c_ sensitivity to VPD (−d*G*_c_/dlnVPD) across studied species as well as under contrasting soil water and *R*_s_ conditions in the urban area.

**Conclusions/Significance:**

We concluded that urban trees show the same hydraulic regulation over response to VPD across varying tree size and environmental conditions and thus tree transpiration could be predicted with appropriate assessment of *G*_cref_.

## Introduction

Establishing urban forest/trees is widely accepted as one of the critical approaches to combat the rapid urbanization associated environmental, ecological, and human health problems [1]–[3]. The potential impact of urban forest/tree development on water resources availability is questioned due to the projected more sever water stress for urban area under global climate change [4], [5] and world-wide reorganization of reduced water yield at watershed scale from increased forest coverage due to land conversion, industry plantation, and forest ecological restoration programs [6], [7]. Our current understanding on forest/tree and water relations under urban environment is very limited [8]. Therefore, it is vital for assessing accurately the role vegetation may play in affecting urban water budget by understanding the eco-physiological response and environmental control of tree water use in urban environments [9].

Practically, tree species selection is of significance for the urban forest development from the water resources management viewpoint as observed species differences in controlling transpiration rate [10]–[12] add variability to the water flux leaving ecosystems and consequently to the hydrological cycle under natural environment [13], [14]. These differences can lead to a substantial spatial heterogeneity of canopy transpiration [15] and the amount of stand transpiration [16], [17]. For example, *Fraxinus excelsior* showed a greater tendency in enhancing transpiration towards the edge than *Quercus robur* did in natural mixed deciduous woodland stands [18]. Annual stand-scale transpiration from bamboo forest is higher than that from the coniferous forests in western Japan [19]. Dynamically, stand transpiration can be significantly enhanced during succession as species shift from woodland oaks and elms to Savanna oaks [20]. Therefore, it is highly probable that species differences in transpiration would influence local hydrology [6], [20]–[24].

Despite the convincing evidence of species effects on transpiration rates, analysis of the relationship between transpiration and tree size or hydrological control [25]–[27] have revealed the convergence in functioning across plant species. Meinzer et al. found that variation in diameter at breast height (DBH) accounted for 91% of the variation in total daily sap flux in the outermost 2 cm of sapwood by comparing 24 different species in a Panamanian rain forest [28]. Thus, the examination of species influence on transpiration is complicated by structural factors such as root, leaf and sapwood area. Regulation of canopy conductance (*G*_c_) exerts a major control over plants transpiration. Stomatal closure is a well known mechanism to regulate plant water status and avoid fatal xylem cavitation under decreasing humidity [29]. Despite the fact that vulnerability to cavitation is highly species specific [30], a synthesis showed that the stomatal response to VPD can be described by a proportionate relationship between the *G*_c_ sensitivity to VPD (−d*G*_c_/dlnVPD or −m) and *G*_c_ at VPD = 1 kPa (*G*_cref_) across 40 species and this relationship was theoretically verified [27]. The theoretical underpinnings suggests that for isohydric species that regulate minimum leaf water potential to prevent excessive cavitation, hydraulic control over transpiration and the ratio between *G*_c_ sensitivity to VPD and *G*_cref_ should be the same despite varying *G*_cref_ between species.

Urban areas tend to exhibit higher air temperature (e.g. “heat island” phenomena), more complex wind turbulence, and increased evaporative demand than the adjacent rural areas due to dense buildings and road system pavement. In addition, urban trees are usually planted in isolation or in rows. These characteristics may induce varied energy partitioning and thus microclimatic difference from natural forest stands. A natural question to ask is “Are there any differences for urban trees from that of natural forest in transpiration control?” as the tree hydraulic control tends to be related to the originating habitat [27]. Therefore, our study was conducted to explore the functional convergence across urban tree. Specifically, our objectives are to examine 1) the influence of tree species and size on transpiration, and 2) the hydraulic control of urban trees over the transpiration in response to VPD as represented by canopy conductance under different environmental conditions.

## Materials and Methods

### Site description and tree selection

The study was conducted in Laodong Park (38°54′N, 121°37′E), Dalian City, Liaoning Province, China. The temperate maritime climate is characterized by mean annual temperature ranging from 8°C–10°C and mean annual precipitation 550–800 mm with 60%–70% in summer.

The study plot is a man-made tree patch located in the north side of the park consisting of *Cedrus deodara* (Roxb) Loud., *Zelkova schneideriana* Hend.-Mazz., *Euonymus bungeanus* Maxim, and *Metasequoia glyptostroboides* Hu et cheng. The trees were originally planted with varying sizes to meet the aesthetic demand. Therefore, the DBH distribution is not the result of growth competition. Due to the restriction from park management, three trees were selected for each species with different DBHs. Trunks of sampled trees were bored to measure the sapwood. It was easy to distinguish the sapwood from heartwood by color difference contrasted by water content (Table 1, also see [31]).

**Table 1:** Characteristics of all sampled trees for sap flow measurement.

Species	DBH(cm)		Projected Crown area (m^2^)		Tree Height (m)		Sapwood area (cm^2^)	
	Mean	SE	Mean	SE	Mean	SE	Mean	SE
*C. deodara*	17.27	3.02	16.56	5.19	7.03	0.93	33.99	8.19
*Z. schneideriana*	13.93	3.11	18.77	4.32	5.30	0.49	18.99	5.84
*M. glyptostroboides*	19.33	4.10	4.80	2.10	11.60	1.06	36.63	14.46
*E. bungeanus*	13.50	2.53	34.96	10.17	5.63	0.48	51.44	12.31

### Sap flow Measurement and Canopy Conductance

Sap flux (*J*_s_) of individual trees was measured continuously from June 25^th^ to October 17^th^ in 2009 using thermal dissipation probes (Dynamax, USA) [32]. A square of 5 cm*5 cm bark at a height of 1.3 m was removed to expose the cambium and the probes were installed. Thirty mm probes were used in all trees except two smaller *Z. schneideriana* trees whose shallow sapwood required the use of 20 mm probes. After the installation, the probes were sealed with silicon foam to prevent rain water infiltration and shielded with aluminum foil to insulate external thermal influences. The output from the probes was recorded as half-hourly average from measurements made at 10 s intervals and stored in a CR1000 data logger (Campbell Scientific, Inc., Logan, UT, USA). The sensors, the heaters and the data loggers were all powered by a 700 mA storage cell. Sap flux can be calculated according to standard calibration for the TDP method based on temperature differences between the two probes [32]:
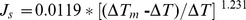
(1)where *J*_s_ is sap flux density (g cm^−2^ s^−1^), Δ*T*_m_ is the maximum temperature difference between sensors during a day (°C), Δ*T*, Temperature difference between sensors at any given time (°C). This equation works well for non-porous species [33], and we acknowledge the existence of limitations when using it for ring-porous species due to non-uniform sap flow [34], [35]. However, given the small sapwood width for our studied trees, we assume the radial velocity gradient is not steep. Moreover, the contact between the probes and non-conducting xylem will give rise to greater errors [34],therefore, the sap flux density of only one depth was examined. Studies show good agreement between sap flux calculated by this equation and other independent transpiration measurements for ring-porous trees [36].

Daily canopy transpiration (*E*_c_, mm d^−1^) then can be calculated as
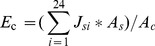
(2)Where *A*_s_ is the sapwood area (cm^2^) and *A_c_* is the crown area (m^2^), *i* stands for the sequence of daily hours..

Canopy conductance (*G*_c_) was calculated by using measured canopy transpiration and Penman-Monteith equation [37]:
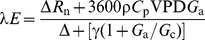
(3)Where λ is the latent heat of vaporization of water (2.39 MJ kg^−1^), Δ is the ratio of the saturated vapor pressure to temperature (kPa °C^−1^), *R*_n_ is the net radiation (MJ m^−2^ h^−1^), estimated from the regression equation 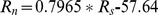 (recommended by Zeppel et al. [37], *R*_s_ is the total radiation, MJ m^−2^ h^−1^), ρ is air density (kg m^−3^), *C*_p_ is the specific heat of air (1.013 MJ kg^−1^ °C^−1^), VPD is the vapor pressure deficit (kPa), γ is the psychrometer constant (0.066 kPa °C^−1^). The constant 3600 is time conversion factor from second to hour. The unit of *E* here is m^3^ m^2^ h^−1^.

### Meteorology and Soil Moisture Content Measurement

Meteorological data were collected using an automatic weather system Weather-Hawk (Campbell Scientific, Logan, UT, USA) mounted at the height of 15 m and 5 m away from the sampled trees. Volumetric soil water content (θ, m^3^ m^−3^) was measured by two sets of ECH2O (Decagon Devices Inc., Pullman, WA, USA). The measurement was made at 25 cm, 50 cm, 75 cm, and 100 cm of soil profile. Relative extractable water (REW, unitless) [38]–[41] was calculated by using averaged θ across layers as
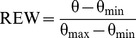
(4)where θ_max_ and θ_min_ are the maximum and the minimum measured θ during the observation period, respectively. The cumulative soil water depletion for each drying cycle was calculated as the difference of profile averaged soil moisture content (θ) between the two consecutive rainfall events.

### Model description

A modified Jarvis-type model (i.e. multiplicative environmental drivers) was used to simulate measured tree transpiration as the parameters in this model are very effective in capturing species differences in responding to the environmental variables [42]. The two most responsible atmospheric factors, solar radiation and vapor pressure deficit (VPD), are used in the model to describe the sap flux variation. The modified Jarvis model is
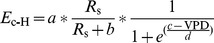
(5)where *E*_c-H_ is hourly canopy transpiration (mm.h^−1^), *R*_s_ total radiation (W m^−2^), and VPD vapor pressure deficit (kPa). Parameter *a* is maximum modeled canopy transpiration under ideal environmental condition. *b* describes light saturation level. *c* and *d* reflect plants response to VPD with *c* being interpreted as VPD level when half of maximum *E*_c-H_ is reached and *d* as the slope between *E*_c-H_ and VPD.

To estimate the average canopy stomatal sensitivity to VPD, we employed the simplified formula used by Oren et al. [27] :
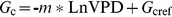
(6)where *G*_c_ is canopy conductance, an estimate of average stomatal conductance over the canopy (mm s^−1^) [43], −m is the slope of *G*_c_ versus LnVPD (i.e. −d *G*_c_/dLnVPD), which quantifies the sensitivity of average canopy stomatal conductance to VPD. *G*_cref_ is reference canopy conductance when VPD = 1 kPa and can be used as surrogate for *G_c_*_max_ [43].

### Statistical analysis

All statistical analysis was performed using SPSS (Version 16.0, Chicago, IL). Curve fitting was run through SigmaPlot (version 10.0, Systat Software, California, USA) and parameters of individual trees were analyzed among species or environmental condition ranks via one-way ANOVA. ANOVA analysis was employed to test the existence of significant differences among groups, and the multiple comparisons of the results were performed by LSD post-hoc test.

In this study, we first tested the assumption that the relationship between −m and *G*_cref_ defined by Eq. (6) follows 0.6 slope [27] across the studied species, and then quantified *G*_c_ responses to θ using boundary line analysis between *G*_cref_ and θ as described by Schäfer et al. [44].

## Results

### Micrometeorology and soil water condition

**Figure 1:**
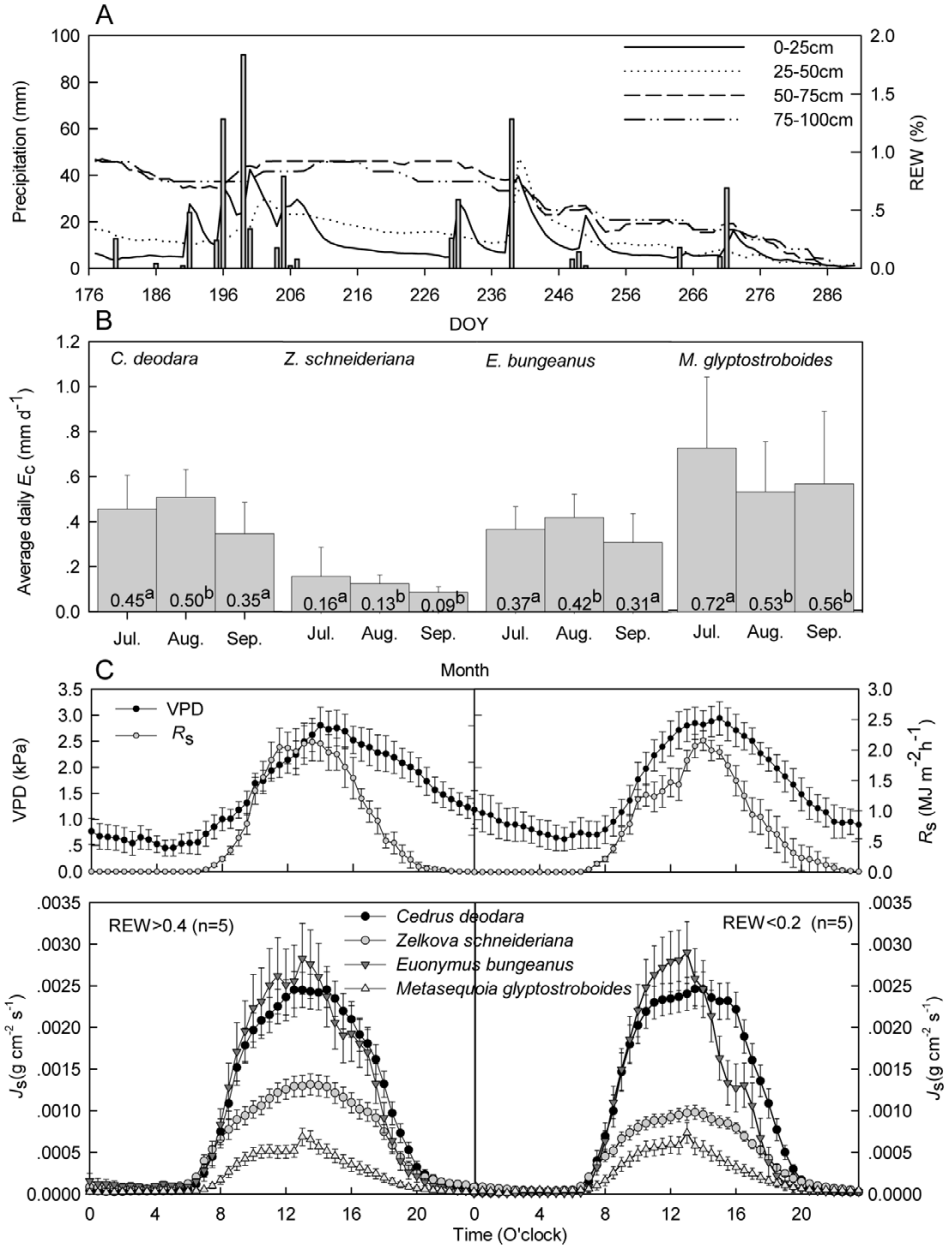
Water supply and transpiration. A: Rainfall and soil water condition during the studied period. B: Comparison of average daily *E*_c_ among different months and species. Significant differences among months within the same species are indicated by upper different lower case letters. Vertical bars stand for S.E.. C: Progression of sap flux density (*J*_s_) under different soil water conditions (REW>0.4 and REW<0.2) in comparison with contemporary VPD and *R*_s_.

Statistics of the averaged daily VPD, daily total solar radiation, and daily air temperature for July, August, and September, 2009 is shown in Table 2. There were significant differences in daily total solar radiation between the three months (*P*<0.001, n = 92, One-way ANOVA) with September lower than the other two. Averaged daily VPD was significantly higher in August than in July (*P* = 0.000, LSD post-hoc test) and September (*P* = 0.024, LSD post-hoc test). Total rainfall was 432.5 mm from July 1^st^ to September 31^st^ with several intensive rainfall events. Soil moisture content in the upper layer depleted more quickly than deeper layers below 50 cm which maintained stable during several dry spells between the rain events (Fig. 1A).

**Table 2:** Statistics of atmosphere variables.

Month	Averaged daily VPD (kPa)	Daily total Solar radiation (MJ.m^−2^)	Averaged daily Temperature (°C)	Monthly Rainfall(mm)
	Mean(S.E.)	Mean(S.E.)	Mean(S.E.)	Sum
July	0.85^a^(0.07)	13.54^a^ (0.99)	23.94^a^ (0.25)	265.25
August	1.33^b^ (0.1)	14.59^a^ (0.77)	25.47^b^ (0.44)	106.75
September	1.05^a^(0.08)	9.67^b^ (0.67)	20.85^c^ (0.29)	60.5

Significant differences (*P*<0.05) among months are indicated by different upper lower case letters.

### Canopy transpiration and its relationship with DBH

Transpiration varied considerably among the individuals both across and within species. The averaged daily total canopy transpiration was different across the species (Fig. 1B). Canopy transpiration of *C*. *deodara* and *E*. *bungeanus* were similar and did not show continuous decline as summer passed by. By contrast, *Z*. *schneideriana* showed decreasing canopy transpiration which was lower than the other species. Similarly, transpiration of *M*. *glyptostroboides* declined throughout the observation period but recovered a little in September from transpiration decrease in August (Fig. 1B). On a daily scale, *J*_s_ of different species followed similar pattern but varied in magnitude under different soil water conditions of similar *R*_s_ and VPD (Fig. 1C).

**Figure 2:**
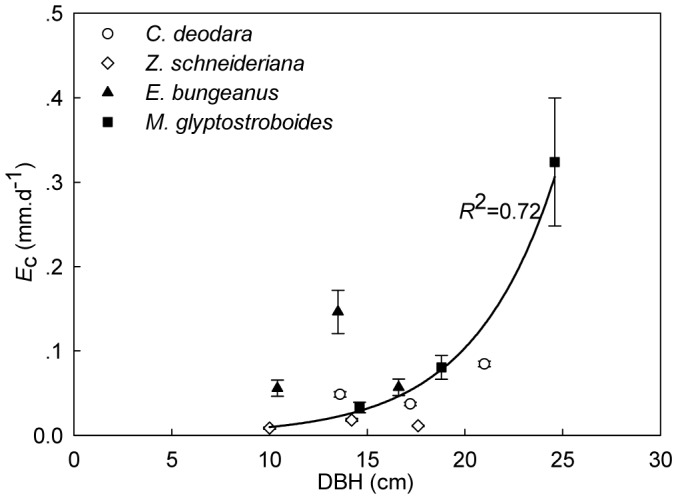
Tree size and transpiration. Relationship between daily canopy transpiration and DBH of individual trees of all investigated species.

Individually, average daily transpiration rate ranged from 0.07 mm d^−1^ by the smallest *E*. *bungeanus* tree to 0.20 mm d^−1^ by the largest *C*. *deodara* tree. It could be partially ascribed to the dependence of water use on tree size (Fig. 2). However, there were considerable species-specific differences in canopy transpiration as indicated by different species within same DBH class. For instance, water transpired by *E*. *bungeanus* (0.195 mm. d^−1^) was nearly 5-fold than that by *Z*. *schneideriana* (0.03 mm d^−1^) as measured for ∼14 cm DBH trees of these two species. When considering the statistical relationship between DBH and *E*_c_, the biggest *M*. *glyptostroboides* triggered an exponential rise (*R* ^2^ = 0.72).

### E_c_ response to environmental drivers

**Figure 3:**
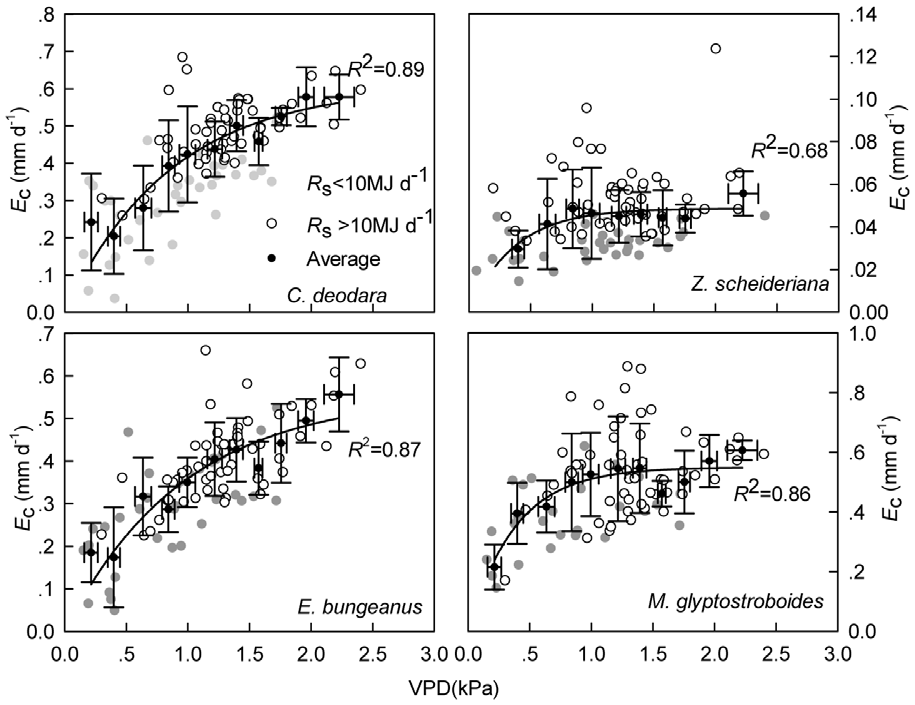
VPD and transpiration. Relationship between daily canopy transpiration in relation to VPD under different solar radiation (*R*_s_) ranks with data on rainy days removed. Black dots represent average *E*_c_ values within every 0.2 kPa rank. If a rank has less than 3 data, this rank is not considered to reduce potential bias from lack of representativeness.

Daily *E*_c_ was closely related to *R*_s_ and VPD (*P*<0.05) and the introduction of other variables did not further normalize the residual distribution. Curve fitting results showed that *E*_c_ saturated at higher VPD and total radiation (*R*_s_) (Fig. 3, 4A). It was also observed that higher *R*_s_ would significantly enhance *E*_c_ under same VPD condition (Fig. 3). However, *E*_c_ under same *R*_s_ condition failed to show significant differences among VPD ranks (*P*>0.05). REW ranks neither affected the relationship between daily *E*_c_ and *R*_s_ nor *E*_c_ and VPD (*P>*0.05).

**Figure 4:**
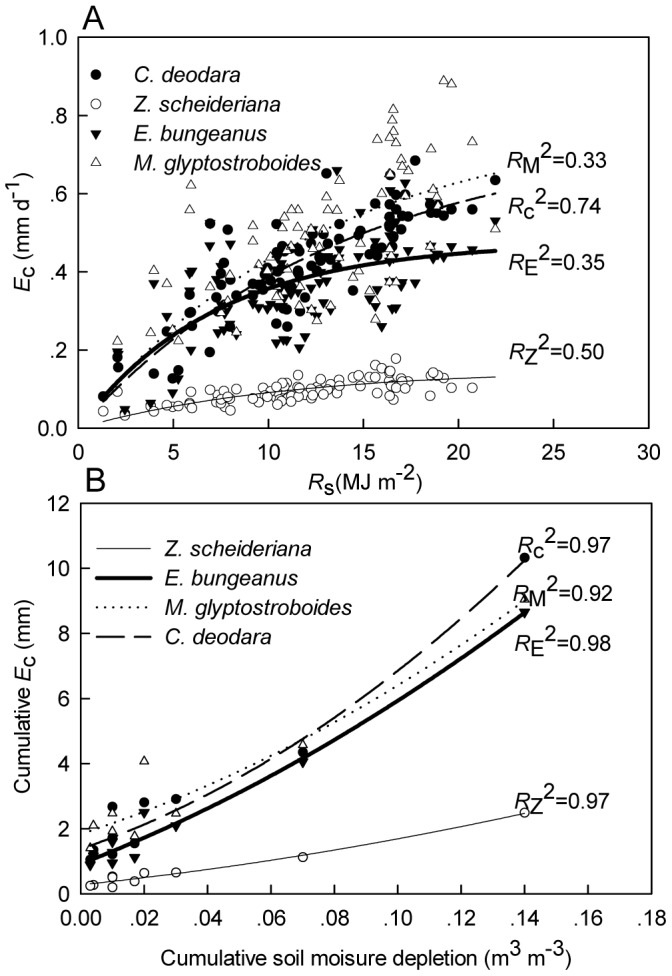
Influences of solar radiation and soil water over transpiration. Daily *E*_c_ in response to *R*_s_ (A) and the relationship between sums of daily transpiration and cumulative soil water depletion for each drying cycle between two consecutive rainfall events (B). The lines are the fitting curves for different species. (A) and (B) share the same legends. *R* ^2^ for each species is denoted with subscripted first letter of the Latin name.

Daily water transpired by trees did not show a significant correlation with REW (*P*>0.40) for plants did not react to concurrent soil water status. However, cumulative daily transpiration was significantly related to the cumulative soil water depletion during each drying cycle (Fig. 4B). When compared according to different REW ranks under similar VPD conditions (*P* = 0.024), diurnal course of transpiration exhibited contrasting patterns (Fig. 1C). The *J*_s_ decline of *E*. *bungeanus* in afternoon was steeper under soil water stress and the *J*_s_ of *Z*. *schneideriana* was suppressed during the whole day. By contrast, no significant reduction of *J*_s_ was observed for *C*. *deodara* and *M*. *glyptostroboides*.

### G_c_ sensitivity

**Figure 5:**
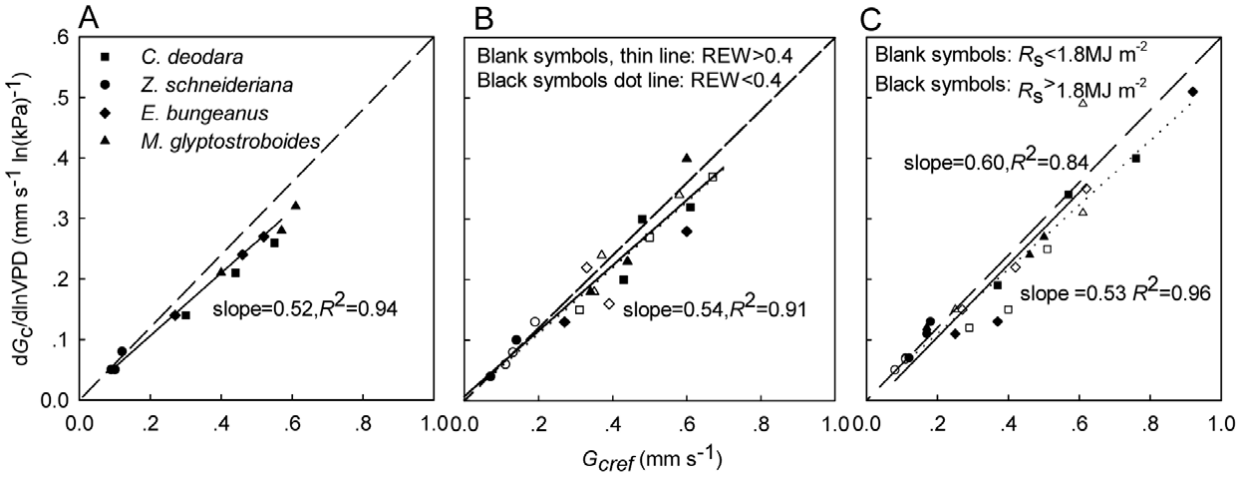
The sensitivity of canopy conductance. Relationship between sensitivity of canopy conductance to VPD (−d*G*_c_/dlnVPD) and canopy conductance at VPD = 1 kPa (*G*_cref_) under (A) all environmental conditions, (B) contrasting REW ranks and (C) different solar radiation (*R*_s_) levels. Data dots were from data in Table 3.

Logarithmic decrease of midday canopy conductance against VPD suggested progressive stomata closure to prevent excessive caviation as the air turned drier across species [31]. There were significant species difference in the *G*_c_ sensitivity to VPD (−d*G*_c_/dlnVPD or −m) and the *G*_cref_ value within each REW and *R*_s_ rank (Table 3). No significant difference was observed for parameters of the same species under contrasting soil water and *R*_s_ ranges, and paired −d*G*_c_/dVPD and *G*_cref_ of different species still followed a strong linear relationship of ∼0.6 slope (Fig. 5). Similar results were also obtained through boundary line analysis of the relationship between *G*_cref_ and REW (data not shown). The lack of significant deviation from a 0.6 slope indicates that all species were isohydric under all environmental conditions.

**Table 3:** Parameters and significance for 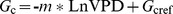 under different soil moisture and radiation conditions.

	General condition^1^		REW<0.4		REW>0.4		*R*_s_<1.8 MJ. m^−2^		*R*_s_>1.8 MJ. m^−2^	
	−m	*G*_cref_	−m	*G*_cref_	−m	*G*_cref_	−m	*G*_cref_	−m	*G*_cref_
*C. deodara*	0.20^a^(0.06)	0.43^a^(0.12)	0.27^a^(0.03)	0.51^a^ (0.05)	0.26^a^(0.06)	0.49^a^(0.18)	0.17^a^(0.07)	0.40^a^(0.11)	0.31^a^(0.11)	0.57^a^(0.19)
*Z. schneideriana*	0.12^b^(0.02)	0.10^b^(0.02)	0.07^b^(0.02)	0.11^b^(0.03)	0.09^b^(0.04)	0.14^b^(0.02)	0.12^b^(0.01)	0.10^b^(0.02)	0.23^b^(0.03)	0.16^b^(0.03)
*E. bungeanus*	0.22^c^(0.07)	0.42^c^(0.13)	0.21^c^(0.11)	0.44^c^(0.13)	0.19^c^(0.02)	0.36^c^(0.02)	0.24^c^(0.10)	0.44^c^(0.17)	0.25^c^(0.22)	0.51^c^(0.35)
*M.glyptostroboides*	0.28^d^(0.06)	0.50^d^(0.09)	0.27^d^(0.06)	0.46^d^(0.07)	0.25^d^(0.05)	0.43^d^(0.07)	0.32^d^(0.17)	0.49^d^(0.21)	0.21^d^(0.08)	0.38^d^(0.18)

Data were given as mean of all sampled trees of the same species and S.E. in parenthesis.

*R*_s_: Solar radiation.

1 : Fitting curves run through *G*_c_ and VPD under entire REW and *R*_s_ range.

a,b,c,d: Significant difference of the parameter among species.

### Model simulation

**Figure 6:**
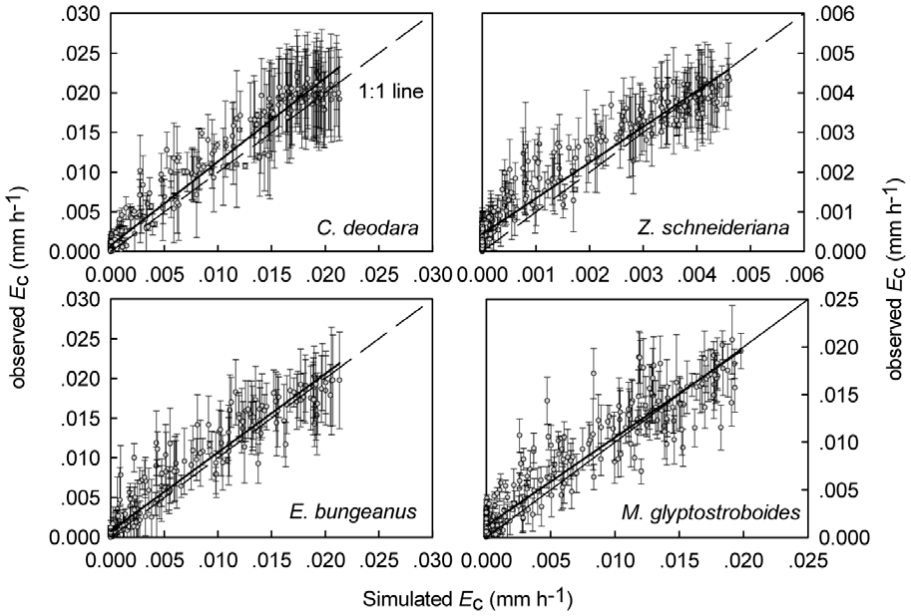
Model simulation. Simulated and observed hourly canopy conductance (*E*_c-H_) for model 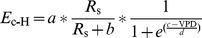 validation using six sampled days in August and September. Observed data were presented as mean of the trees within the same species±SD.

Average *E*_c-H_ of all three trees within the same species from 30 days was included for model calibration and 6 days were randomly chosen for validation. Model performances were satisfactory for both calibration (see *R^2^_ad_*_j_>0.89 in Table 4) and validation as the simulated half-hourly *E*_c-H_ agreed well with observed values (Fig. 6). Model parameters showed significant differences in maximum transpiration (parameter *a*) (*P*<0.001, one-way ANOVA) and in light saturation level (parameter *b*, *P* = 0.004) among species. For species response to VPD, significant differences were found for VPD corresponding to the level at half maximum transpiration (parameter *c*, *P* = 0.024, one-way ANOVA) and the slope between *E*_c-H_ and VPD (parameter *d, P* = 0.035, one-way ANOVA) across species. Moreover, the level of response to VPD remained stable across days as *c* and *d* did not show significant differences among the replicate days (*P* = 0.552 for *c* and 0.621 for *d*) for each species

**Table 4:** Calibrated parameters for 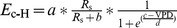 for species.

Species	*a* (mm.h^−1^)		*b* (MJ.m^−2^.h^−1^)		*c* (kPa)		*d* (kPa)		*R* ^2^_adj_	
	Mean	SE	Mean	SE	Mean	SE	Mean	SE	Mean	SE
*Cedrus deodara*	0.035^a^	0.001	1.00^a^	0.20	0.26^a^	0.06	1.65^a^	0.1	0.96	0
*Zelkova schneideriana*	0.009^b^	0.004	1.88^b^	0.02	0.41^b^	0.11	1.45^b^	0.07	0.92	0.03
*Euonymus bungeanus*	0.065^c^	0.001	3.09^c^	0.03	1.41^c^	0.12	1.57^c^	0.09	0.88	0.11
*Metasequoia glyptostroboides*	0.050^d^	0.001	1.76^d^	0.03	1.72^d^	0.32	1.78^d^	0.16	0.92	0.04

Significant differences across species (*P*<0.05) are indicated with upper lower case letters.

## Discussion

### Influences from tree size and Species on Transpiration

Comparable to general conclusion drawn from previous studies that tree size plays an overwhelming role in influencing stand water use [9], [25], [26], [45], [46], our study observed a positive relationship between transpiration and DBH independent of species (Fig. 2). The significant relationship between transpiration and DBH is attributable to the positive relationship between DBH and sapwood area which directly transports sap flow [47]. A sigmoidal increase portrayed the relation between water use and DBH among 18 angiosperm species in tropical forest [45]. Similarly, tree size has been found in some studies to be more important than species in determining transpiration [28] which is explained by pipe model theory [48]. It is a robust non-destructive method to estimate biomass [49]–[52]. According to the theory, the amount of leaves on a tree is supported by a proportionate cross sectional area of a bundle of pipes with equal hydraulic conductance. Therefore, the greater the living biomass the tree has the more sapwood area it has. However, the theory also indicates that the relationship between leaf area and sapwood cross area is species-specific. Consequently, the species effects on transpiration also can be strong even with changing tree size. In our study, the variation of average daily transpiration by four species ranged from 0.07 to 0.20 mm. d^−1^. This can also be ascribed to species specific canopy conductance, such as *E*. *bungeanus* in comparison with other species within ∼14 cm DBH class. Such appreciable species-specific differences in transpiration suggested potential large influence of species composition at scales larger than stands [22].

Tree size can be a determinant factor on transpiration of highly structured stands [53], but the relationship may be more depend on the stand DBH composition. For example, Pataki et al. did not observe significant relationship between tree size and transpiration in urban areas and the author attributed it to limited range of DBH distribution which was common in the urban green spaces [8]. In our study the exponential increase between *E*_c_ and DBH was triggered by one big *M*. *glyptostroboides* without which the relationship changed to linear and the *R* ^2^ decreased significantly (Fig. 2). It shows the influence of DBH composition, especially the large ones, on the relationship with transpiration. In urban green space, the tree size is usually unbalanced to represent a continuous DBH range, so it is not reliable to predict water use of urban trees using its relationship with tree size.

### Response to Environmental variables and Seasonal Transpiration Patterns

All four species showed saturation of tree transpiration to high VPD and *R*_s_ and this phenomenon has been widely reported [54]–[57]. When the canopy is well-coupled with the atmosphere [31], plants exert effective stomatal control over transpiration to regulate the minimum water potential according to VPD and it was also predictable from the 0.6 relationship between *G*_cref_ and −d*G*_c_/dlnVPD. Therefore, *E*_c_ plateaus with increasing VPD as exhibited in this study. The *E*_c_ saturation to increasing *R*_s_ can be ascribed to the fact that stomata are fully opened at certain level of irradiance [58] and energy is not limiting to transpiration for these trees, and therefore do not react to higher radiation levels.

The influence of soil moisture on transpiration has not been consistent among studies [59]–[62]. In this study, daily *E*_c_ did not show significant correlation with soil moisture content. At the sub-daily time scales, mesic tree species show a greater response to light and VPD with soil moisture playing a limited role except under conditions where root to soil moisture resistance is high [63]. However, soil moisture content is an important environmental driver for tree transpiration for longer time scales (Fig. 4B) [64], [65].

Varied pattern of species transpiration in response to seasonal weather progression (Fig. 1B) was also observed. Due to *J*_s_ sensitivity to soil water stress (Fig. 1C), the transpiration of *Z*. *schneideriana* decreased along the progression of rain fall reduction through the months. However, the declining trend of transpiration of *M*. *glyptostroboides* should be ascribed to variation of VPD because of the sensitive *G*_c_ response to VPD by this species and high *G*_cref_ (Fig. 5A). Therefore, transpiration by this species declined as VPD increased in August and recovered in September as VPD declined. In addition to the low rainfall, the high transpiration of *C*. *deodara* and *E*. *bungeanus* in August may contribute to the build up cavitation in the xylem and subsequently reduce transpiration thereafter [30].

### Sensitivity to VPD

On a plot basis, trees of different species observed same hydraulic control (Fig. 5) and species differences in *G*_cref_ led to varied magnitude of canopy transpiration across species (Fig. 1B, 3). In our study, even though −d*G*_c_/dlnVPD and *G*_cref_ showed significant difference among species, their ratios for all these four species converged to ∼0.6 as reported [27], [66]. The changes of *G*_cref_ and −d*G*_c_/dlnVPD were along the 0.6 slope under varying REW and solar radiation conditions and no significant bias existed in relation to tree size similar to what was found by Ewers et al [43] for one tree species exposed to manipulated soil moisture and nutrients. The interpretation is that isohydric regulation of water loss through *G*_c_ response to VPD [27], [67] is the same among the four species regardless of varying tree sizes and environmental conditions, while the *G*_cref_ is species-specific. This relationship also means that *G*_c_ sensitivity to VPD [67] and the transpiration of urban trees can be reliably predicted from *G*_cref_ [68], [69].

Since environmental conditions did not influence the relationship between -d*G*_c_/dlnVPD and *G*_cref_, *G*_cref_ can serve as an efficient indicator of species differences. The constant relationship between −d*G*_c_/dlnVPD and *G*_cref_ reflects an isohydric stomatal control which protects the xylem from developing runaway cavitation by guaranteeing safe water potential [63]. Species difference in *G*_cref_ may be related to xylem characteristics which tolerate cavitation. In addition to the partitioning of xylem resistance as well as aquaporin activity [70], [71], xylem anatomic differences were most commonly related to species' cavitation tolerances [30], [72]–[74]. Species difference in xylem tolerance to cavitation was explicitly revealed through the daily *J*_s_ rate under contrasting REW conditions (Fig. 1C). With larger diameter xylem vessels, ring-porous trees were less tolerant to negative water potential and xylem cavitation than diffuse-porous species due to intrinsic vulnerability of large diameter conduits to cavitation [30], [75], such as *Z*. *schneideriana* versus *E*. *bungeanus* in our case. This is further reflected by the decreased *Js* of *Z*. *schneideriana* under water stress (Fig. 1C). However *E*. *bungeanus* could afford the same magnitude of transpiration under water stress as under no water stress during first half of the day. By contrast, *C. deodara* and *M*. *glyptostroboides* were able to maintain normal transpiration rate. That is probably because as gymnosperm species, they have tracheids of small diameter and strong cell wall, features that are resistant to cavitation.

Sensitivity of canopy conductance to VPD has great implications for the survival of trees in urban landscape. Trees of high canopy conductance at low VPD shows higher sensitivity to VPD [27], [76], such as trees with higher *G*_cref_ in this study. Active control over *G*_c_ enables isohydric species to maximize carbon assimilation under low VPD and avoid the risk of runaway cavitation under atmospheric or soil drought [77]. Therefore, these species are suitable for urban environment where unpredictable local change is widespread [78]. Artificial activity produces extra thermal energy, which could be complicated temporally and spatially due to air turbulence caused by building arrangement [79]. Another factor comes from irrigation scope and intensity. Without irrigation, soil water condition is more likely to be stressed because of prevented rainfall percolation from pavements and enhanced soil evaporation in unpaved area. Although effective in *G*_c_ control that guarantees tree survival, species with low maximum canopy conductance through the entire VPD range have less advantage in competitive situation, such as multi-species urban forest in summer, because lower *G*_c_ does not favor carbon assimilation for growth in competition with high *G*_c_ species. Low carbon assimilation might be potentially harmful to tree survival through a major drought event from the perspective of water safety [80]. As a result, species with different water use strategies will be differently affected by shifts of the frequency, duration, and intensity of drought [81].

### Implication of Species Differences on Urban Transpiration

Species differences tended to be revealed through the relationship between transpiration and environmental drivers. In our study, the modified Jarvis-type model described the environmental control over plants transpiration well (Fig. 6) and model parameters captured species differences in responding to the environment influences (Table 4). Parameter differences, i.e. species differences in responding to environmental factors, were also found among species by studies in tropical forest [42] and in Philippines [53]. Our study also showed increased expression of species differences under water stress (Fig. 1C).

Under urban environment, species differences in transpiration and response to the environment may cause spatial difference in hydraulic redistribution among green space even within small scope of area. Also, it may influence the effect of micro-meteorology modulation by vegetation. Even with such appreciable species differences, it is possible to predict urban canopy transpiration using their shared hydraulic control character. Unlike natural setting where transpiration can be assessed using the relationship with DBH, the influence of DBH on canopy transpiration is undermined because of the limited DBH distribution in the city. Our results recommend using the ∼0.6 relationship between *−*d*G*_c_/dLnVPD and *G*_cref_. As *G*_c_ under 1 kPa can be accurately tested via proper measurements including tree-based or remote sensed techniques, *G*_c_, and thus canopy transpiration, can be assessed at any scale through concurrent VPD.

## Conclusion

Species differences were found in an urban environment in the response of transpiration to environmental drivers including light, soil moisture and vapor pressure deficit and in the control of tree size on transpiration. Despite significant species differences, all species showed declining *G*_c_ against increasing VPD, and the theoretical −d*G*_c_ */*dlnVPD to *G*_cref_ ratio of ∼0.6 was observed across studied species and under contrasting soil water and *R*_s_ conditions in the urban area. We, therefore, concluded that urban trees show isohydric regulation of minimum leaf water potential as reflected in their response to VPD and that transpiration can be predicted with appropriate assessment of *G*_c_ at VPD = 1 kPa.

## References

[1] Murphy DJ, Hall MH, Hall CAS, Heisler GM, Stehman SV, et al (2011) The relationship between land cover and the urban heat island in northeastern Puerto Rico. Int J Climatol 31: 1222–1239.

[2] Hedblom M, Söderström B (2010) Landscape effects on birds in urban woodlands: an analysis of 34 Swedish cities. J Biogeogr 37: 1302–1316.

[3] Heisler GM, Grant RH (2000) Ultraviolet radiation in urban ecosystems with consideration of effects on human health. Urban Ecosyst 4: 193–229.

[4] Walther G-R, Post E, Convey P, Menzel A, Parmesan C, et al (2002) Ecological responses to recent climate change. Nature 416: 389–395. 11919621 10.1038/416389a

[5] Allen CD, Macalady AK, Chenchouni H, Bachelet D, McDowell N, et al (2010) A global overview of drought and heat-induced tree mortality reveals emerging climate change risks for forests. For Ecol Manage 259: 660–684.

[6] Jackson RB, Jobbagy EG, Avissar R, Roy SB, Barrett DJ, et al (2005) Trading water for carbon with biological sequestration. Science 310: 1944–1947. 16373572 10.1126/science.1119282

[7] Zhang L, Dawes WR, Walker GR (2001) Response of mean annual evapotranspiration to vegetation changes at catchment scale. Water Resour Res 37: 701–708.

[8] Pataki DE, McCarthy HR, Litvak E, Pincetl S (2010) Transpiration of urban forests in the Los Angeles metropolitan area. Ecol Appl 21: 661–677. 10.1890/09-1717.1 21639035

[9] Meinzer FC, Andrade JL, Goldstein G, Holbrook NM, Cavelier J, et al (1999) Partitioning of soil water among canopy trees in a seasonally dry tropical forest. Oecologia 121: 293–301. 28308316 10.1007/s004420050931

[10] Franks PJ (2004) Stomatal control and hydraulic conductance, with special reference to tall trees. Tree Physiol 24: 865–878. 15172837 10.1093/treephys/24.8.865

[11] Abril M, Hanano R (1998) Ecophysiological responses of three evergreen woody Mediterranean species to water stress. Acta Oecologica 19: 377–387.

[12] Benyon RG, Marcar NE, Theiveyanathan S, Tunningley WM, Nicholson AT (2001) Species differences in transpiration on a saline discharge site. Agric Water Manage 50: 65–81.

[13] Ford CR, Hubbard RM, Kloeppel BD, Vose JM (2007) A comparison of sap flux-based evapotranspiration estimates with catchment-scale water balance. Agric For Meteorol 145: 176–185.

[14] Oren R, Pataki D (2001) Transpiration in response to variation in microclimate and soil moisture in southeastern deciduous forests. Oecologia 127: 549–559. 28547493 10.1007/s004420000622

[15] Hölscher D, Koch O, Korn S, Leuschner C (2005) Sap flux of five co-occurring tree species in a temperate broad-leaved forest during seasonal soil drought. Trees-Struct Funct 19: 628–637.

[16] Kaufmann MR (1985) Annual transpiration in subalpine forests: Large differences among four tree species. For Ecol Manage 13: 235–246.

[17] Licata JA, Pypker TG, Weigandt M, Unsworth MH, Gyenge JE, et al (2011) Decreased rainfall interception balances increased transpiration in exotic ponderosa pine plantations compared with native cypress stands in Patagonia, Argentina. Ecohydrol 4: 83–93.

[18] Herbst M, Roberts JM, Rosier PTW, Taylor ME, Gowing DJ (2007) Edge effects and forest water use: A field study in a mixed deciduous woodland. For Ecol Manage 250: 176–186.

[19] Komatsu H, Onozawa Y, Kume T, Tsuruta K, Kumagai To, et al (2010) Stand-scale transpiration estimates in a Moso bamboo forest: II. Comparison with coniferous forests. For Ecol Manage 260: 1295–1302.

[20] Asbjornsen H, Tomer MD, Gomez-Cardenas M, Brudvig LA, Greenan CM, et al (2007) Tree and stand transpiration in a Midwestern bur oak savanna after elm encroachment and restoration thinning. For Ecol Manage 247: 209–219.

[21] van Dijk AIJM, Keenan RJ (2007) Planted forests and water in perspective. For Ecol Manage 251: 1–9.

[22] Mackay DS, Ahl DE, Ewers BE, Gower ST, Burrows SN, et al (2002) Effects of aggregated classifications of forest composition on estimates of evapotranspiration in a northern Wisconsin forest. Global Change Biol 8: 1253–1265.

[23] Iida Si, Tanaka T, Sugita M (2005) Change of interception process due to the succession from Japanese red pine to evergreen oak. J Hydrol 315: 154–166.

[24] Bittner S, Talkner U, Krämer I, Beese F, Hölscher D, et al (2010) Modeling stand water budgets of mixed temperate broad-leaved forest stands by considering variations in species specific drought response. Agric For Meteorol 150: 1347–1357.

[25] Cienciala E, Kucera J, Malmer A (2000) Tree sap flow and stand transpiration of two Acacia mangium plantations in Sabah, Borneo. J Hydrol 236: 109–120.

[26] Meinzer FC, James SA, Goldstein G (2004) Dynamics of transpiration, sap flow and use of stored water in tropical forest canopy trees. Tree Physiol 24: 901–909. 15172840 10.1093/treephys/24.8.901

[27] Oren R, Sperry JS, Katul GG, Pataki DE, Ewers BE, et al (1999) Survey and synthesis of intra- and interspecific variation in stomatal sensitivity to vapour pressure deficit. Plant Cell Environ 22: 1515–1526.

[28] Meinzer FC, Goldstein G, Andrade JL (2001) Regulation of water flux through tropical forest canopy trees: do universal rules apply? Tree Physiol 21: 19–26. 11260820 10.1093/treephys/21.1.19

[29] Sperry JS (2000) Hydraulic constraints on plant gas exchange. Agric For Meteorol 104: 13–23.

[30] Bush SE, Pataki DE, Hultine KR, West AG, Sperry JS, et al (2008) Wood anatomy constrains stomatal responses to atmospheric vapor pressure deficit in irrigated, urban trees. Oecologia 156: 13–20. 18270747 10.1007/s00442-008-0966-5

[31] Chen L, Zhang Z, Li Z, Tang J, Caldwell P, et al (2011) Biophysical control of whole tree transpiration under an urban environment in Northern China. J Hydrol 402: 388–400.

[32] Granier A (1987) Evaluation of transpiration in a Douglas-fir stand by means of sap flow measurements. Tree Physiol 3: 309–320. 14975915 10.1093/treephys/3.4.309

[33] Bush SE, Hultine KR, Sperry JS, Ehleringer JR (2010) Calibration of thermal dissipation sap flow probes for ring- and diffuse-porous trees. Tree Physiol 30: 1545–1554. 21112973 10.1093/treephys/tpq096

[34] Clearwater MJ, Meinzer FC, Andrade JL, Goldstein G, Holbrook NM (1999) Potential errors in measurement of nonuniform sap flow using heat dissipation probes. Tree Physiol 19: 681–687. 12651324 10.1093/treephys/19.10.681

[35] Steppe K, De Pauw DJW, Doody TM, Teskey RO (2010) A comparison of sap flux density using thermal dissipation, heat pulse velocity and heat field deformation methods. Agric For Meteorol 150: 1046–1056.

[36] Granier A, Anfodillo T, Sabatti M, Cochard H, Dreyer E, et al (1994) Axial and radial water flow in the trunks of oak trees: a quantitative and qualitative analysis. Tree Physiol 14: 1383–1396. 14967611 10.1093/treephys/14.12.1383

[37] Zeppel MJB, Macinnis-Ng CMO, Yunusa IAM, Whitley RJ, Eamus D (2008) Long term trends of stand transpiration in a remnant forest during wet and dry years. J Hydrol 349: 200–213.

[38] Gartner K, Nadezhdina N, Englisch M, Cermak J, Leitgeb E (2009) Sap flow of birch and Norway spruce during the European heat and drought in summer 2003. For Ecol Manage 258: 590–599.

[39] Kumagai T, Saitoh TM, Sato Y, Morooka T, Manfroi OJ, et al (2004) Transpiration, canopy conductance and the decoupling coefficient of a lowland mixed dipterocarp forest in Sarawak, Borneo: dry spell effects. J Hydrol 287: 237–251.

[40] Palomo MJ, Moreno F, Fernández JE, Díaz-Espejo A, Girón IF (2002) Determining water consumption in olive orchards using the water balance approach. Agric Water Manage 55: 15–35.

[41] Tognetti R, Giovannelli A, Lavini A, Morelli G, Fragnito F, et al (2009) Assessing environmental controls over conductances through the soil-plant-atmosphere continuum in an experimental olive tree plantation of southern Italy. Agric For Meteorol 149: 1229–1243.

[42] O'Brien JJ, Oberbauer SF, Clark DB (2004) Whole tree xylem sap flow responses to multiple environmental variables in a wet tropical forest. Plant Cell Environ 27: 551–567.

[43] Ewers BE, Oren R, Johnsen KH, Landsberg JJ (2001) Estimating maximum mean canopy stomatal conductance for use in models. Can J For Res 31: 198–207.

[44] Schäfer KVR, Oren R, Tenhunen JD (2000) The effect of tree height on crown level stomatal conductance. Plant Cell Environ 23: 365–375.

[45] Meinzer FC, Bond BJ, Warren JM, Woodruff DR (2005) Does water transport scale universally with tree size? Funct Ecol 19: 558–565.

[46] Wullschleger SD, Hanson PJ, Todd DE (2001) Transpiration from a multi-species deciduous forest as estimated by xylem sap flow techniques. For Ecol Manage 143: 205–213.

[47] Macfarlane C, Bond C, White DA, Grigg AH, Ogden GN, et al (2010) Transpiration and hydraulic traits of old and regrowth eucalypt forest in southwestern Australia. For Ecol Manage 260: 96–105.

[48] Shinozaki K, Yoda K, Hozumi K, Kira T (1964) A quantitative theory of plant form-the pipe model theory,I.Basic analysis. Jpn J Ecol 14: 97–104.

[49] Valentine HT, Mäkelä A (2012) Modeling forest stand dynamics from optimal balances of carbon and nitrogen. New Phytol 194: 961–971. 22463713 10.1111/j.1469-8137.2012.04123.x

[50] Berninger F, Nikinmaa E, Sievänen R, Nygren P (2000) Modelling of reserve carbohydrate dynamics, regrowth and nodulation in a N2-fixing tree managed by periodic prunings. Plant Cell Environ 23: 1025–1040.

[51] Yoneda T, Tamin R, Ogino K (1990) Dynamics of aboveground big woody organs in a foothill dipterocarp forest, West Sumatra, Indonesia. Ecol Res 5: 111–130.

[52] Ogawa K, Adu-Bredu S, Yokota T, Hagihara A (2010) Leaf biomass changes with stand development in hinoki cypress (*Chamaecyparis obtusa* [Sieb. et Zucc.] Endl.). Plant Ecolog 211: 79–88.

[53] Dierick D, Hölscher D (2009) Species-specific tree water use characteristics in reforestation stands in the Philippines. Agric For Meteorol 149: 1317–1326.

[54] Granier A, Huc R, Barigah ST (1996) Transpiration of natural rain forest and its dependence on climatic factors. Agric For Meteorol 78: 19–29.

[55] Zhang H, Morison J, Simmonds L (1999) Transpiration and water relations of poplar trees growing close to the water table. Tree Physiol 19: 563–573. 12651530 10.1093/treephys/19.9.563

[56] Hogg EH, Black TA, den Hartog G, Neumann HH, Zimmermann R, et al (1997) A comparison of sap flow and eddy fluxes of water vapor from a boreal deciduous forest. J Geophys Res 102: 28929–28937.

[57] Hogg EH, Hurdle PA (1997) Sap flow in trembling aspen: implications for stomatal responses to vapor pressure deficit. Tree Physiol 17: 501–509. 14759823 10.1093/treephys/17.8-9.501

[58] Yunusa IAM, Aumann CD, Rab MA, Merrick N, Fisher PD, et al (2010) Topographical and seasonal trends in transpiration by two co-occurring *Eucalyptus* species during two contrasting years in a low rainfall environment. Agric For Meteorol 150: 1234–1244.

[59] Lagergren F, Lindroth A (2002) Transpiration response to soil moisture in pine and spruce trees in Sweden. Agric For Meteorol 112: 67–85.

[60] Lundblad M, Lindroth A (2002) Stand transpiration and sapflow density in relation to weather, soil moisture and stand characteristics. Basic Appl. Ecol 3: 229–243.

[61] Wallace J, McJannet D (2010) Processes controlling transpiration in the rainforests of north Queensland, Australia. J Hydrol 384: 107–117.

[62] Huang Y, Li X, Zhang Z, He C, Zhao P, et al (2011) Seasonal changes in Cyclobalanopsis glauca transpiration and canopy stomatal conductance and their dependence on subterranean water and climatic factors in rocky karst terrain. J Hydrol 402: 135–143.

[63] Sperry JS, Adler FR, Campbell GS, Comstock JP (1998) Limitation of plant water use by rhizosphere and xylem conductance: results from a model. Plant Cell Environ 21: 347–359.

[64] Llorens P, Poyatos R, Latron J, Delgado J, Oliveras I, et al (2010) A multi-year study of rainfall and soil water controls on Scots pine transpiration under Mediterranean mountain conditions. Hydrol Processes 24: 3053–3064.

[65] Fisher RA, Williams M, Da Costa AL, Malhi Y, Da Costa RF, et al (2007) The response of an Eastern Amazonian rain forest to drought stress: results and modelling analyses from a throughfall exclusion experiment. Global Change Biol 13: 2361–2378.

[66] Morison JI, Gifford RM (1983) Stomatal sensitivity to carbon dioxide and humidity: a comparison of two C_3_ and two C_4_ grass species. Plant Physiol 71: 789–796. 16662909 10.1104/pp.71.4.789 PMC1066124

[67] Ewers BE, Gower ST, Bond-Lamberty B, Wang CK (2005) Effects of stand age and tree species on canopy transpiration and average stomatal conductance of boreal forests. Plant Cell Environ 28: 660–678.

[68] Novick K, Oren R, Stoy P, Juang J-Y, Siqueira M, et al (2009) The relationship between reference canopy conductance and simplified hydraulic architecture. Adv Water Resour 32: 809–819.

[69] Mackay DS, Samanta S, Nemani RR, Band LE (2003) Multi-objective parameter estimation for simulating canopy transpiration in forested watersheds. J Hydrol 277: 230–247.

[70] Nardini A, Salleo S, Raimondo F (2003) Changes in leaf hydraulic conductance correlate with leaf vein embolism in *Cercis siliquastrum* L. Trees-Struct Funct 17: 529–534.

[71] Zwieniecki MA, Brodribb TJ, Holbrook NM (2007) Hydraulic design of leaves: insights from rehydration kinetics. Plant Cell Environ 30: 910–921. 17617819 10.1111/j.1365-3040.2007.001681.x

[72] Markesteijn L, Poorter L, Bongers F, Paz H, Sack L (2011) Hydraulics and life history of tropical dry forest tree species: coordination of species' drought and shade tolerance. New Phytol 191: 480–495. 21477008 10.1111/j.1469-8137.2011.03708.x

[73] Linton MJ, Sperry JS, Williams DG (1998) Limits to water transport in Juniperus osteosperma and Pinus edulis: implications for drought tolerance and regulation of transpiration. Funct Ecol 12: 906–911.

[74] Pratt RB, Jacobsen AL, Ewers FW, Davis SD (2007) Relationships among xylem transport, biomechanics and storage in stems and roots of nine Rhamnaceae species of the California chaparral. New Phytol 174: 787–798. 17504462 10.1111/j.1469-8137.2007.02061.x

[75] Li YY, Sperry JS, Taneda H, Bush SE, Hacke UG (2008) Evaluation of centrifugal methods for measuring xylem cavitation in conifers, diffuse- and ring-porous angiosperms. New Phytol 177: 558–568. 18028295 10.1111/j.1469-8137.2007.02272.x

[76] Yong JWH, Wong SC, Farquhar GD (1997) Stomatal responses to changes in vapour pressure difference between leaf and air. Plant Cell Environ 20: 1213–1216.

[77] Katul GG, Palmroth S, Oren RAM (2009) Leaf stomatal responses to vapour pressure deficit under current and CO_2_-enriched atmosphere explained by the economics of gas exchange. Plant Cell Environ 32: 968–979. 19389053 10.1111/j.1365-3040.2009.01977.x

[78] Liu W, You H, Dou J (2009) Urban-rural humidity and temperature differences in the Beijing area. Theor Appl Climatol 96: 201–207.

[79] Rizk AA, Henze GP (2010) Improved airflow around multiple rows of buildings in hot arid climates. Energy Build 42: 1711–1718.

[80] McDowell NG (2011) Mechanisms linking drought, hydraulics, carbon metabolism, and vegetation mortality. Plant Physiol 155: 1051–1059. 21239620 10.1104/pp.110.170704 PMC3046567

[81] West AG, Hultine KR, Sperry JS, Bush SE, Ehleringer JR (2008) Transpiration and hydraulic strategies in a pinon-juniper woodland. Ecol Appl 18: 911–927. 18536252 10.1890/06-2094.1

